# Sleep promotes lasting changes in selective memory for emotional scenes

**DOI:** 10.3389/fnint.2012.00108

**Published:** 2012-11-21

**Authors:** Jessica D. Payne, Alexis M. Chambers, Elizabeth A. Kensinger

**Affiliations:** ^1^Department of Psychology, University of Notre DameNotre Dame, IN, USA; ^2^Boston CollegeChestnut Hill, MA, USA

**Keywords:** emotional memory formation, emotional memory enhancement, sleep, sleep and memory, memory consolidation, memory, emotion

## Abstract

Although we know that emotional events enjoy a privileged status in our memories, we still have much to learn about how emotional memories are processed, stored, and how they change over time. Here we show a positive association between REM sleep and the selective consolidation of central, negative aspects of complex scenes. Moreover, we show that the placement of sleep is critical for this selective emotional memory benefit. When testing occurred 24 h post-encoding, subjects who slept soon after learning (24 h Sleep First group) had superior memory for emotional objects compared to subjects whose sleep was delayed for 16 h post-encoding following a full day of wakefulness (24 h Wake First group). However, this increase in memory for emotional objects corresponded with a decrease in memory for the neutral backgrounds on which these objects were placed. Furthermore, memory for emotional objects in the 24 h Sleep First group was comparable to performance after just a 12 h delay containing a night of sleep, suggesting that sleep soon after learning selectively stabilizes emotional memory. These results suggest that the sleeping brain preserves in long-term memory only what is emotionally salient and perhaps most adaptive to remember.

Emotional memories form the core of our personal histories. They shape our personalities by representing our greatest achievements and our worst defeats, mark the milestones in our changing lives, and figure prominently in anxiety and mood disorders (see Payne et al., 2004; Kensinger, 2009; Brewin et al., 2010 for review). Although we know that emotional experiences enjoy a privileged status in our memories, being better remembered than most neutral events, researchers are still learning how memories for emotional events are processed and stored (McGaugh, 2004; LaBar and Cabeza, 2006), and how they change over time (Payne and Kensinger, 2010).

The notion that memories are not static, but rather form gradually, dates back to Muller and Pilzecker (1900), who coined the term “memory consolidation” (reviewed in McGaugh, 2000; Dudai, 2004). Memory consolidation is (at least) a two-stage process, including molecular and cellular events that support the strengthening of synapses over minutes and hours (“cellular consolidation”), and system-wide changes that occur in the hours and days following learning (“systems consolidation”). Growing evidence suggests that the offline brain state of sleep provides ideal conditions for memory consolidation, particularly on a systems level (reviewed by Stickgold, 2005; Ellenbogen et al., 2006; Walker and Stickgold, 2006; Diekelmann and Born, 2010; Payne, 2011). For example, neural activation patterns seen during daytime task training are reactivated during subsequent sleep in both rats and humans (Diekelmann and Born, 2010; Girardeau and Zugaro, 2011), and such reactivations can lead to performance enhancements the following day (Peigneux et al., 2004; Rasch et al., 2007).

Several studies demonstrate that sleep preferentially enhances emotional episodic memories over neutral ones. When presented with both negative arousing and neutral information as part of the same learning experience, subjects who sleep between training and test preferentially consolidate negative over neutral narratives (Wagner et al., 2001, 2006), pictures (Hu et al., 2006), and components of scenes (Payne and Kensinger, 2010 for review). For example, Payne et al. (2008) showed that when presented with scenes consisting of either neutral (e.g., a chipmunk) or negative arousing (e.g., a snake) objects placed on neutral backgrounds (e.g., a forest), participants who slept selectively consolidated memory for the emotional objects, while memory for the accompanying backgrounds deteriorated. Thus, as compared to a 30-min control group, those who slept showed selective memory benefits for the emotional objects, but showed no corresponding benefit for their backgrounds or for either the objects or the backgrounds composing neutral scenes. Those who stayed awake, on the other hand, showed poorer memory for all elements as compared to those tested after a 30-min delay. These results are interesting for two reasons. First, they contribute to a growing literature suggesting that the effects of emotion on memory are intensified during sleep (see Walker, 2009; Payne, 2011; Payne and Kensinger, 2011 for review). Second, the sleeping brain, rather than simply influencing the consolidation of all recently encountered information, appears to select for consolidation only what is most emotionally salient about experience and perhaps most relevant to future goals (see also Payne et al., 2009; Payne and Kensinger, 2010). The idea of goal relevance is particularly apropos in this task given that the way emotion affects memory (i.e., narrowing vs. broadening) depends on a person's current goal (Levine and Edelstein, 2009); a negative or threatening stimulus, such as the snake in the forest described above, might trigger a survival goal, which might in turn trigger a narrowing of one's focus onto the threatening object (the snake) at the expense of the background (the forest) in a way the neutral object (the chipmunk) might not.

Although there is currently much debate about which features of sleep are most important for memory consolidation, converging lines of evidence suggest that rapid eye movement sleep (REM) critically modulates memory for highly arousing emotional information. Hennevin et al. (1998) demonstrate that aversive conditioning in rodents is followed by increases in REM sleep that often continue until the task is mastered (Hennevin et al., 1995). Further, depriving rats of REM sleep can lead to performance deficits in avoidance tasks, particularly if the deprivation occurs within so-called “REM-windows” (Smith and Butler, 1982; Smith, 1995; Smith and Rose, 1996).

Preliminary human research suggests a similarly beneficial effect of REM sleep on emotional memory consolidation. Wagner et al. (2001) found that 3 h of late night, REM-rich sleep (but not 3 h of early night slow-wave-rich sleep) facilitated memory for negative arousing narratives, an effect that could still be observed years later when the subjects were re-contacted for a second memory test (Wagner et al., 2006), and REM sleep theta activity has been positively correlated with memory for emotional pictures (Nishida et al., 2009; see Walker, 2009, for review). Sleep's importance to emotional processing is also suggested by the emotionally charged and vivid dreams of REM sleep, which may utilize elements of past experiences to simulate and practice for threatening future situations (Levin and Nielsen, 2009; Payne and Kensinger, 2011). Moreover, affective disorders such as depression are characterized not only by changes in emotional memory (e.g., Hamilton and Gotlib, 2008), but also by marked changes in REM sleep architecture (Berger and Riemann, 1993; Pillai et al., 2011).

Each of these lines of evidence is interesting given that the amygdala and hippocampus are among the most active brain regions during REM sleep (Maquet et al., 1996), perhaps signaling interactions between these regions as they selectively process emotional memories offline. While neutral episodic memories rely on the hippocampus and adjacent structures for their processing, emotional episodic memories receive a special boost from the amygdala, which modulates activity in the hippocampus to preferentially influence emotional memory formation. Interestingly, a recent study showed greater activity in these regions and greater connectivity among them following sleep compared to wakefulness (Payne and Kensinger, 2011; see also Sterpenich et al., 2009).

Although the tendency to remember emotional over neutral events can be observed after short delays, at times emotional arousal has a more potent influence on memory across long delays (24 h) than short ones (e.g., Kleinsmith and Kaplan, 1963; Walker and Tarte, 1963; Sharot et al., 2007; Sharot and Yonelinas, 2008), and damage to the amygdala disproportionately affects retention of emotional information over long delays while having less influence on short-term retention (Phelps et al., 1997, 1998; LaBar and Phelps, 1998). These results may be in part explained by neurohormonal modulation of amygdala-hippocampus interactions that occur “offline,” post-encoding (Payne, 2011 for review). For example, the memory modulation hypothesis (McGaugh, 2004) argues that greater long-term memory for emotional over neutral information reflects the neuromodulatory influence of the amygdala on hippocampus-based consolidation processes, via stress hormone engagement. In this issue, for example, Echterhoff and Wolf (2012) show that the combination of viewing a highly arousing event coupled with a subsequent increase in stress produces an increase in bias to remember central aspects of the event in an eyewitness memory task. Notably, the release of the stress hormone cortisol parallels REM episodes throughout the night, and reaches its diurnal zenith during late night REM–rich sleep (reviewed in Payne and Kensinger, 2011).

Clearly, there is good reason for emotional memories to be preferentially consolidated during sleep, perhaps especially during REM. However, in spite of converging evidence from the lines of work described above, this idea is not without its problems. Most problematic are interference arguments that provide compelling alternative explanations for sleep-based consolidation effects (Wixted, 2004; Mednick et al., 2011). To date, most of the studies to directly examine the relationship between sleep (vs. wakefulness) and emotional memory formation are confounded by the amount of interference encountered during the waking delay interval. For example, both the Hu et al. (2006) and Payne et al. (2008) studies demonstrated that a 12 h period containing nocturnal sleep selectively benefits memory for negative arousing stimuli relative to an equivalent period of daytime wakefulness. An interference account would argue that sleep, rather than conferring an active neurobiological benefit on these memories, merely provided a passive and temporary shield against interfering stimulation because it is a time of reduced sensory input (Wixted, 2004).

Because this “nocturnal sleep vs. daytime wake” design (e.g., see the 12 h Wake and 12 h Sleep conditions in Figure 1, labeled “a”) is open to interference criticisms, Wagner et al. (2001) used a split-night procedure to demonstrate that emotional memories benefit from 3 h of late night REM-rich sleep, relative to 3 h of early slow-wave-rich sleep, or equivalent periods of wakefulness during the night. Although this finding is not easy to reconcile with an interference account, it is subject to circadian and sleep deprivation confounds. Encoding and retrieval processes may vary as a function of circadian phase in ways that are not easily revealed through task performance, and there is also a possibility that consolidation processes are modulated by circadian time, rather than by REM-sleep *per se*. Moreover, the wake control groups may have performed poorly because they were sleep deprived during the first or second half of the night.

**Figure 1:**
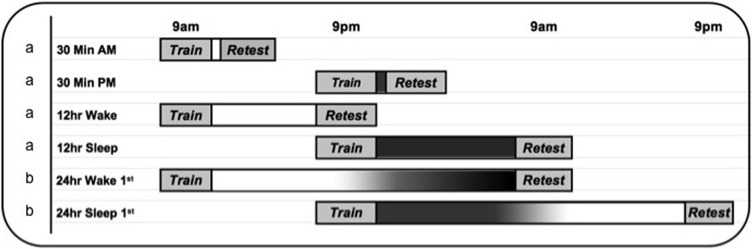
**Experimental timeline**. The 30 min (*n* = 40), 12 h wake (*n* = 24) and 12 h sleep (*n* = 24) groups (labeled “a”) are from Payne et al. (2008). Note: Because there were no differences in performance in the 30 min groups (30 min am, *n* = 20 and 30 min pm, *n* = 20), they were combined into a single 30 min variable for analyses reported in the current paper. The overnight polysomnography study reported here (Experiment 1, *n* = 27) follows the 12 h Sleep protocol (labeled “a”), with the addition of overnight sleep monitoring in the laboratory. The 24 h delay study (Experiment 2, labeled “b”) follows the 24 h Wake 1st (*n* = 22) and 24 h Sleep 1st (*n* = 22) protocols.

Given these confounds, we investigated the influence of sleep on emotional memory formation via a third method: Following the incidental encoding of negative and neutral scenes in the morning or evening, memory was tested following a 24 h delay interval. Time spent sleeping and awake were thus equated (see the 24 h Wake First and 24 h Sleep First conditions in Figure 1, labeled “b”). By an interference account, sleep directly following learning should merely post-pone the deleterious effects of interference that would ensue upon subsequent waking. Memory performance should therefore be equivalent regardless of whether sleep comes soon after learning, or after a day of wakefulness. Alternatively, if memory processing continues on a normal, or perhaps facilitated, level during sleep, performance should be better when sleep comes shortly after learning than when it comes later in the retention interval, despite equal time spent awake during which interference would be expected to occur. A consolidation account would argue for this latter possibility because key events of the consolidation process occur in the minutes and hours following learning (McGaugh, 2000); thus, sleeping soon after encoding should help stabilize the memories and protect them from subsequent interference.

To examine how the positioning of sleep would affect emotional memory formation across a 24 h delay, we presented subjects with neutral or negatively arousing objects (e.g., an intact car or a wrecked car) on a neutral background (e.g., a street), and later tested recognition memory separately for the objects and backgrounds (see Figure 2). This task consistently reveals an “emotional memory trade-off” following brief (e.g., 30 min) time delays (Kensinger et al., 2007). Such trade-offs are said to occur when negatively arousing objects are better remembered than neutral objects, yet the neutral backgrounds associated with negative objects are remembered more poorly than similar backgrounds presented with neutral objects (Figure 2). A real world example of this trade-off is the weapon-focus effect, where victims vividly remember an assailant's weapon but have poor memory for peripheral aspects of the scene (Stanny and Johnson, 2000).

**Figure 2:**
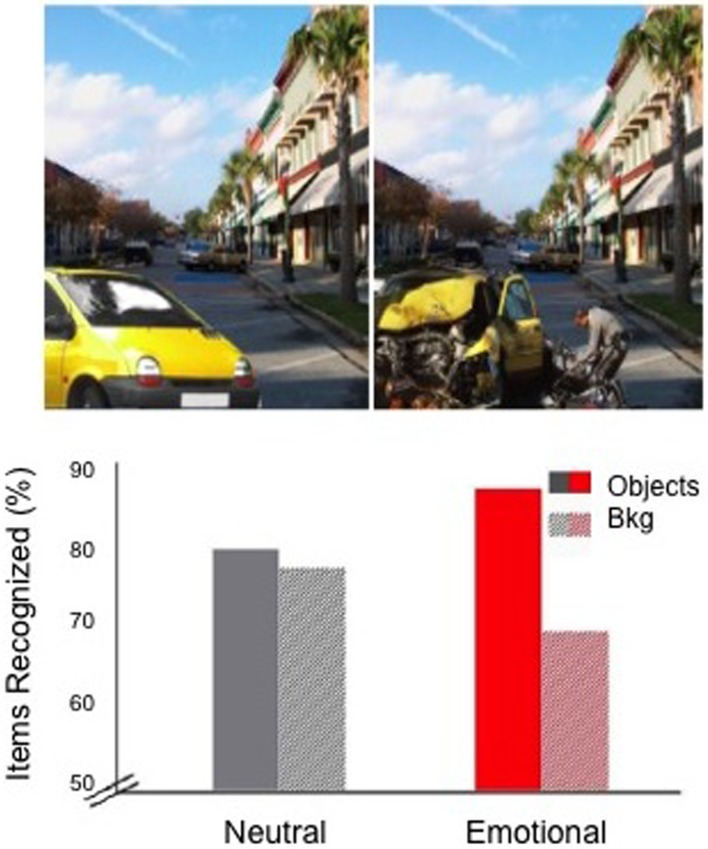
**The emotional memory trade-off effect**. **Top**—Sample stimuli with the identical neutral backgrounds and either a neutral (left) or negative emotional (right) object in the foreground; **Bottom**—Emotional trade-off (following a 30 min delay, from Payne et al., 2008) is seen on right as increased recognition of emotional objects with impaired recognition of neutral backgrounds. Figure adapted from Payne and Kensinger (2010).

Such trade-offs are thought to be produced at encoding, because attention during memory acquisition is focused on the emotionally salient central aspects of the scene at the expense of the neutral features of the background, an effect supported by an eye-tracking study presented in this issue (Niu et al., 2012). However, the emotional memory trade-off is also clearly influenced by post-encoding consolidation processes, as the magnitude of the effect is larger after a delay that includes sleep (Payne et al., 2008). Thus, preliminary evidence suggests that the discrete components of emotional scenes (objects, backgrounds) continue to undergo qualitatively different processing post-encoding. Little is known, however, about how the different components of emotional memories continue to be processed and transformed over longer periods of time following sleep. For example, it is unclear how long such trade-offs last in memory, whether memory for objects and backgrounds remain stable over time, or continue to diverge, and whether the initial positioning of sleep is important for these effects.

The current study had several goals. In the first experiment, we asked whether preferential memory for emotional objects following a night of sleep would be positively correlated with time spent in REM sleep. In the second experiment, we examined whether such preferential emotional remembering would persist across a longer delay of 24 h. Here we also examined the impact of sleep's placement on memory for the components of scenes in two conditions, comparing a condition where sleep occurred shortly after learning to a condition where sleep did not occur until after a day of wakefulness. Importantly, time spent awake and thus subject to interference was equated in these two 24 h delay conditions. While an interference account would predict no differences between the two conditions, a consolidation account would predict a lasting change in the trade-off effect, and perhaps a magnification of it if sleep triggers changes that continue throughout the following day.

## Methods

### Participants

In the first experiment, which was designed to polysomnographically (PSG) examine the overnight sleep stage correlates of selective emotional memory consolidation, students from the University of Notre Dame and Boston College (*n* = 27) participated for payment (age 18–25). In the second study, designed to examine patterns of memory performance across the longer delay (24 h), 44 college students (age 18–22) from Boston College participated for course credit or payment. All subjects slept for >7 h between training and test (*M* = 8.2 for the overnight PSG group, *M* = 8.0 h for the sleep first group, and *M* = 7.5 h for the wake first group; *t* > 0.30, *ns*), and for 7.1 h on average the night prior to the experiment (*M* = 7.3 h for the overnight PSG group, *M* = 7.1 h for the sleep first group, and *M* = 7.0 h for the wake first group; *t* > 0.90, *ns*). All participants were native English speakers with normal or corrected-to-normal vision. Participants reported no history of psychiatric or sleep disorders, nor were they taking medications that affect the central nervous system or sleep architecture.

### Materials

Stimuli in both experiments consisted of negative arousing or neutral objects placed on neutral backgrounds to create realistic scenes (Kensinger et al., 2007; Payne et al., 2008). The scenes were crafted by placing one of a pair of negative arousing objects (e.g., one of two crashed cars) or neutral objects (e.g., one of two intact cars) against one of a pair of neutral backgrounds (e.g., one of two streets). The pairs of objects and backgrounds were selected such that the items of a pair shared the same verbal label (e.g., both were intact cars) but differed in perceptual feature (e.g., color, shape, size, and orientation). Each scene thus consisted of an object (either neutral or negative in valence) placed on a background (which was always neutral). For example, a neutral scene might consist of an avocado (a neutral object) placed on a countertop (a neutral background), while a negative scene might consist of a spider (a negative object) on a countertop (a neutral background). By varying the object type (neutral or negative), the object version (one of two paired objects), and the background version (one of two paired backgrounds), eight versions of the 64 scenes were created.

Objects and backgrounds were selected from a group of stimuli that had been previously rated for arousal and valence using 7-point scales (Kensinger et al., 2006). Negative objects were all given arousal ratings of 5–7 (with high scores indicating an exciting or agitating image and low scores indicating a calming or soothing image) and valence ratings lower than 3 (with lower scores indicating a negative image and high scores indicating a positive image). Neutral objects and backgrounds were rated as non-arousing (with arousal scores lower than 4) and neutral (with valence scores between 3 and 5; see Kensinger et al., 2007 for more details about the rating procedures).

### Procedure

Participants in the first, overnight PSG, study arrived at the sleep laboratory between the hours of 9 and 10 pm. Electrodes were applied while they watched a non-arousing video. Sleep was recorded with Grass/Telefactor/Comet polysomnography systems. The montage included electrooculography (EOG), electromyography (EMG), and EEG leads (F3, F4, C3, Cz, C4), with each electrode referenced to the contralateral mastoid. Sleep data were scored according to the standards of Rechtschaffen and Kales (1968). Following PSG hookup, participants viewed the stimuli (between 10:15 and 11:30 pm) prior to going to bed. After a full night of sleep, and at least 30 min after awakening (to allow for recovery from sleep inertia), recognition memory was tested (between 7 and 9 am). A summary of sleep measures is provided in Table 1.

**Table 1:** **Sleep parameters for subjects in overnight sleep study**.

**Sleep parameter**	**Mean time (min) ± SEM**	**% Total sleep time ± SEM**
Total sleep time	435 ± 12	−
Wake after sleep onset	28 ± 4	−
Sleep latency	12 ± 2	−
Stage 1	23 ± 2	5.3 ± 0.5
Stage 2	228 ± 8	52.3 ± 1.4
Stage 3	30 ± 2	6.9 ± 0.5
Stage 4	60 ± 4	14.0 ± 0.9
SWS (Stages 3 + 4)	90 ± 4	20.7 ± 0.9
REM	94 ± 5	21.6 ± 1.1

An overview of the amount of time spent in various stages of sleep (Sleep Parameters) for the participants in the overnight PSG study. Time is given in both mean time in bed and percentage of the total time asleep. Note: All measures are in minutes. Sleep Latency = latency to sleep onset (first epoch of sleep). S1–S4, Stages 1–4; SWS, Slow Wave Sleep; REM, Rapid Eye Movement sleep.

In the second study, participants were randomly assigned to the “Sleep First” condition (*n* = 22), or the “Wake First” condition (*n* = 22). Subjects in the Sleep First condition viewed the stimuli between the hours of 7 and 9 pm and were tested ~24 h later, again between the hours of 7 and 9 pm. Subjects in the Wake First condition viewed the stimuli between the hours of 9 and 11 am and were tested ~24 h later, again between the hours of 9 and 11 am.

All participants studied a set of 64 scenes (32 with a negative arousing object on a neutral background and 32 with a neutral object on a neutral background) for 5 s each. The version of the scene shown during the study phase was counterbalanced across participants. Once a given scene was removed from the screen, participants were asked to indicate whether they would want to approach or move away from the scene if they were to encounter it in real life, a procedure used to ensure deep encoding in this otherwise incidental learning task. Responses were made by button press, using the numbers 1–7, with “1” indicating that they would move closer, “7” indicating that they would move away, and “4” indicating that they would stay at the same distance.

After the relevant delay period, subjects performed an unexpected, self-paced recognition test. They viewed objects and backgrounds separately and one at a time (i.e., subjects never saw the scenes in their entirety at test). Some of the objects and backgrounds presented were identical to those that had been previously studied (*same)*, others were the alternate version of the object or background pair and thus differed from the studied version in perceptual features but not verbal label (*similar*), and some were new objects or backgrounds that had not been studied (*new*). Participants saw either the *same* or the *similar* version of an object or background on the recognition test, never both versions. Each object and background presented on the screen was accompanied by a question (e.g., “Did you see a spider?”). Participants responded “same” if the answer to the question was “yes” and if the object or background presented was the exact match to what had been viewed during the study phase. Participants responded “similar” if the object or background shared the same verbal label, but was not an exact match to a studied component. If the answer to the question was “no,” participants responded “new,” indicating that the object or background had not been seen during the study phase. All responses were made on the keyboard, with “i” indicating “same/identical,” “s” indicating “similar,” and “n” indicating “new.”

The recognition task included 32 *same* objects (16 negative, 16 neutral), 32 *similar* objects (16 negative, 16 neutral), 32 *new* objects (16 negative, 16 neutral), 32 *same* backgrounds (16 previously shown with a negative object, 16 previously shown with a neutral object), 32 *similar* backgrounds (16 previously shown with a negative object, 16 previously shown with a neutral object), and 32 *new* backgrounds.

### Data analysis

As in other studies requiring subjects to make same/similar distinctions at retrieval (Garoff et al., 2005; Kensinger et al., 2006), we considered “same” responses to *same* items to reflect memory for the visual details of the studied object or background (“specific recognition”). However, this is a highly stringent measure of memory accuracy, particularly in light of the long delay used here. Thus, we also examined memory by a less stringent “overall recognition” measure (“same” + “similar” response to *same* items, see Kensinger et al., 2007). Consistent with previous studies using this measure (e.g., Kensinger et al., 2007; Payne et al., 2008), *same* items given either a “same” or a “similar” (and not a “new”) response were considered to reflect memory for at least some aspects of the studied item, because subjects have to remember at least that a given type of object or background had been studied (i.e., that they had seen some kind of avocado or some kind of countertop)—otherwise, they would have indicated that the item was “new.” Both overall and specific recognition scores were computed separately for the central object (negative or neutral) and for the background on which objects were presented (always neutral). Although false alarms (“same” responses to *new* items) were relatively low (less than 14% in all cases), and did not differ between groups on any measure, all recognition scores were corrected for false alarms. By examining memory performance across 24 h in the “Sleep First” and “Wake First” conditions, we were able to examine how the passage of time influenced memory depending on the placement of sleep.

## Results

### Is memory for emotional objects associated with REM sleep?

Our first goal was to determine whether selective emotional memory was specifically associated with REM sleep. As in Payne et al. (2008), we found clear evidence for the emotional memory trade-off following the 12 h overnight delay. The 2 (Object valence: Negative, Neutral) × 2 (Scene component: Object, Background) repeated measures ANOVA revealed a significant interaction between Object valence and Scene component, both for specific recognition, [*F*_(1, 26)_ = 26.7, *p* < 0.001], and overall recognition [*F*_(1, 26)_ = 23.6, *p* < 0.001]. While specific recognition of negative objects was significantly better than for neutral objects, [*t*_(26)_ = 5.9, *p* < 0.001], memory for backgrounds that had contained these negative objects was impaired relative to backgrounds that had contained neutral objects [*t*_(26)_ = 2.9, *p* = 0.007]. The same was true for overall recognition memory [*t*_(26)_ = 3.3, *p* = 0.003 and *t*_(26)_ = 4.1, *p* < 0.001, respectively].

**Figure 3:**
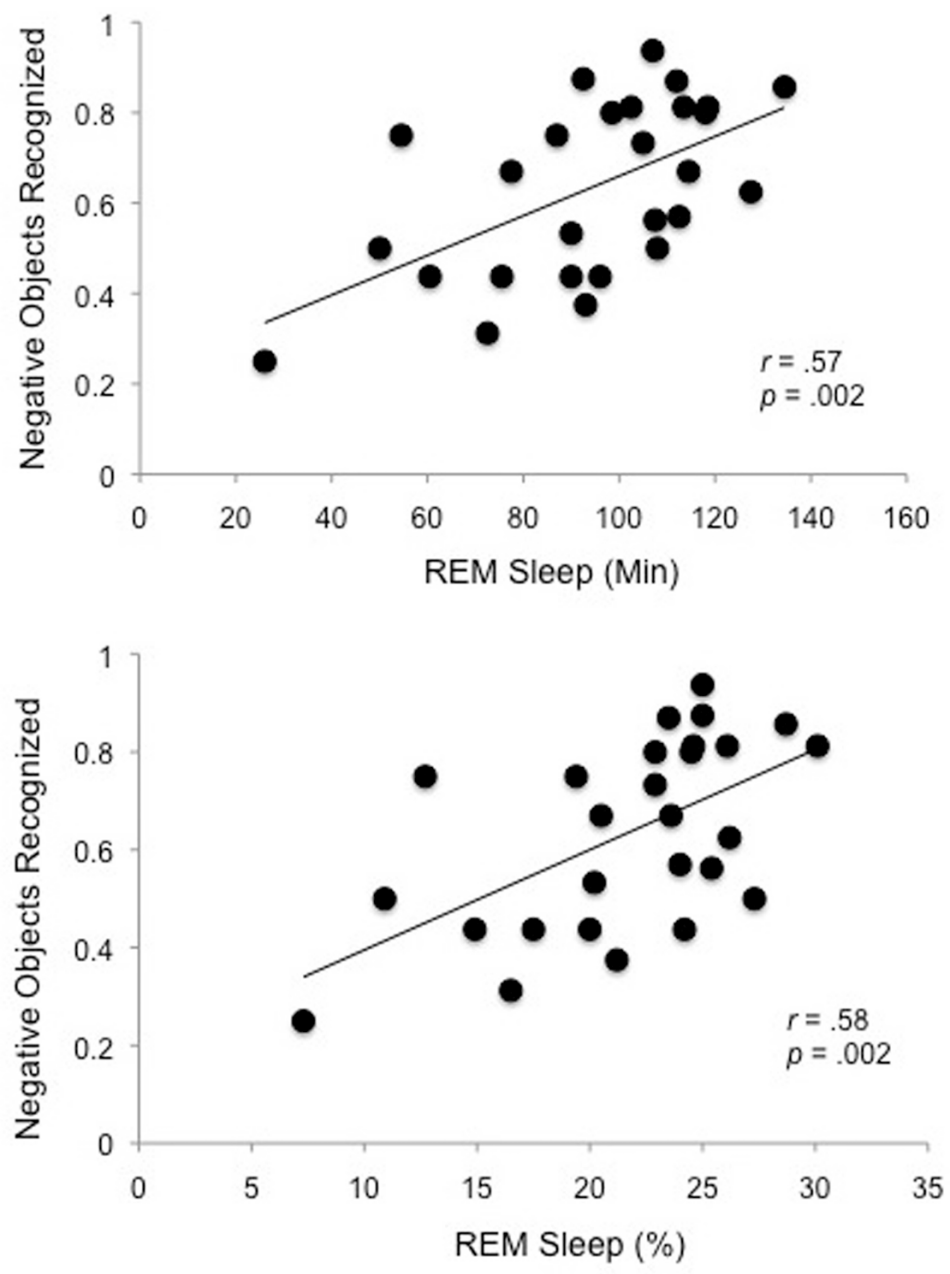
**REM sleep correlations**. Specific recognition of negative objects is selectively positively correlated with time spent in REM sleep (REM Sleep Min) and with percent of the night spent in REM sleep (REM Sleep %).

As predicted, Pearson's *r* correlations revealed that overnight memory for emotional objects was positively correlated with both the total amount of time spent in REM and percent of total sleep time spent in REM (REM%) (Figure 3). This was the case for specific recognition (“same” responses), [REM, *r*_(27)_ = 0.57, *p* = 0.002; REM%, *r*_(27)_ = 0.58, *p* = 0.002] as well as overall recognition (“same” + “similar” responses), [REM *r*_(27)_ = 0.54, *p* = 0.003; REM%, *r*_(27)_ = 0.52, *p* = 0.006]. The specificity of this effect is highlighted by the fact that no other sleep stage (stage 1, 2, SWS), or total sleep time, correlated with emotional object memory, nor did any sleep stage correlate with any of the other memory measures (neutral objects or backgrounds that were associated with either negative or neutral objects).

### Does the emotional memory trade-off persist across a 24 h delay?

**Figure 4:**
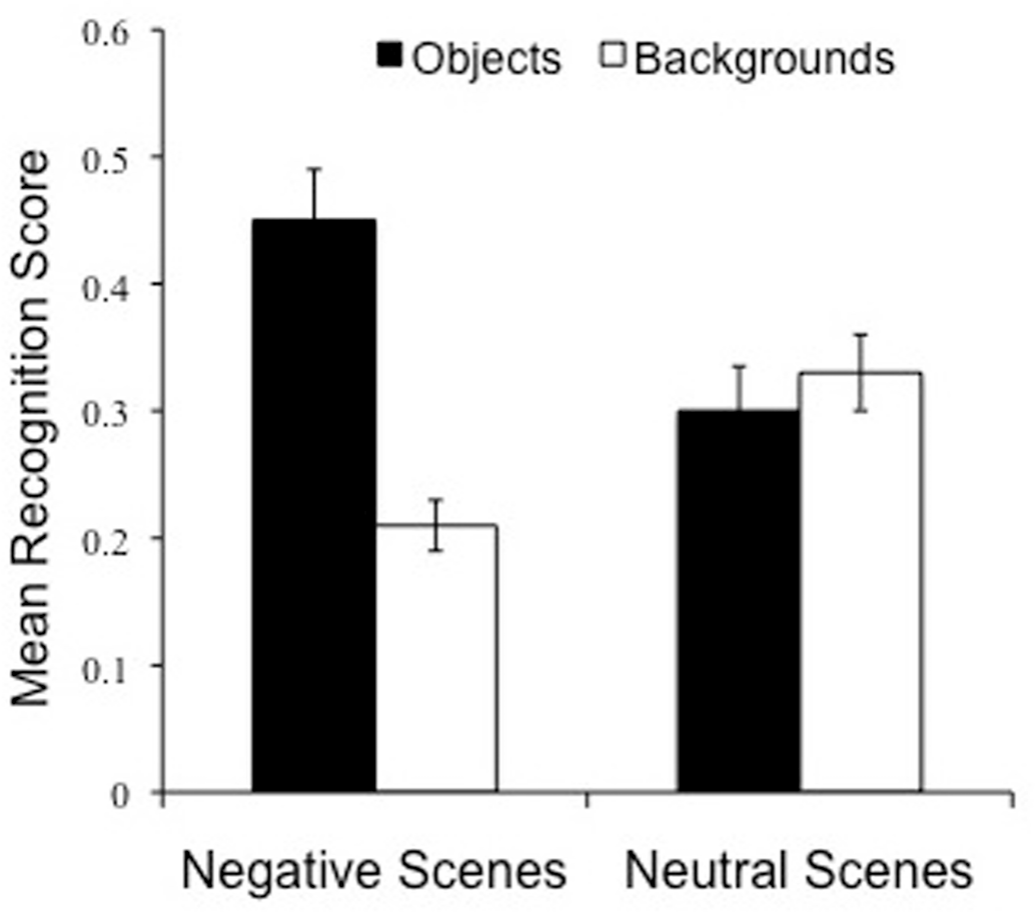
**Twenty-four hours delay memory performance overall**. The emotional memory trade-off effect is preserved following a 24 h delay. Note that object memory is superior for negative, compared to neutral, scenes (*p* < 0.001), while scene backgrounds are more poorly remembered if they are paired with negative, compared to neutral, objects (*p* < 0.001).

Our next goal was to examine whether there would be evidence for the emotional memory trade-off 24 h post-encoding, or rather this long delay would weaken or eliminate it completely. We thus conducted a 2 (Object valence: Negative, Neutral) × 2 (Scene component: Object, Background) repeated measures ANOVA on specific recognition. As predicted, there was a highly significant interaction between Object valence and Scene component, [*F*_(1, 42)_ = 60.1 *p* < 0.001], which confirms the existence of the trade-off 24 h post-encoding (Figure 4). While negative object memory was significantly better than neutral object memory, [*t*_(43)_ = 5.6, *p* < 0.001], memory for backgrounds that had contained these negative objects was impaired relative to backgrounds that had contained neutral objects [*t*_(43)_ = 3.8, *p* < 0.001]. In addition, although objects and backgrounds were recognized at similar rates within neutral scenes, [*t*_(43)_ = 0.7, *p* = 0.48, *ns*] (Figure 4, right), objects were significantly better recognized than backgrounds within negative scenes, [*t*_(43)_ = 4.4, *p* < 0.001] (Figure 4, left). The Valence by Scene component interaction also emerged in overall recognition, [*F*_(1, 42)_ = 56.9, *p* < 0.001]. Again, while negative object memory was significantly better than neutral object memory, [*t*_(43)_ = 5.7, *p* < 0.001], memory for backgrounds that had contained negative objects was impaired relative to backgrounds containing neutral objects [*t*_(43)_ = 3.3, *p* = 0.002]. Moreover, although objects and backgrounds were recognized at similar rates within neutral scenes, [*t*_(43)_ = 0.5, *p* > 0.60, *ns*], objects were significantly better recognized than backgrounds within negative scenes, [*t*_(43)_ = 6.8, *p* < 0.001]. These findings confirm and extend the well-documented trade-off for the central and peripheral components of emotional scenes seen after brief time delays (Kensinger et al., 2007; Payne et al., submitted), and after a 12 h delay (Payne et al., 2008; Payne and Kensinger, 2011), revealing that the effect is still present 24 h after study.

### The trade-off effect in the sleep first vs. wake first conditions

**Figure 5:**
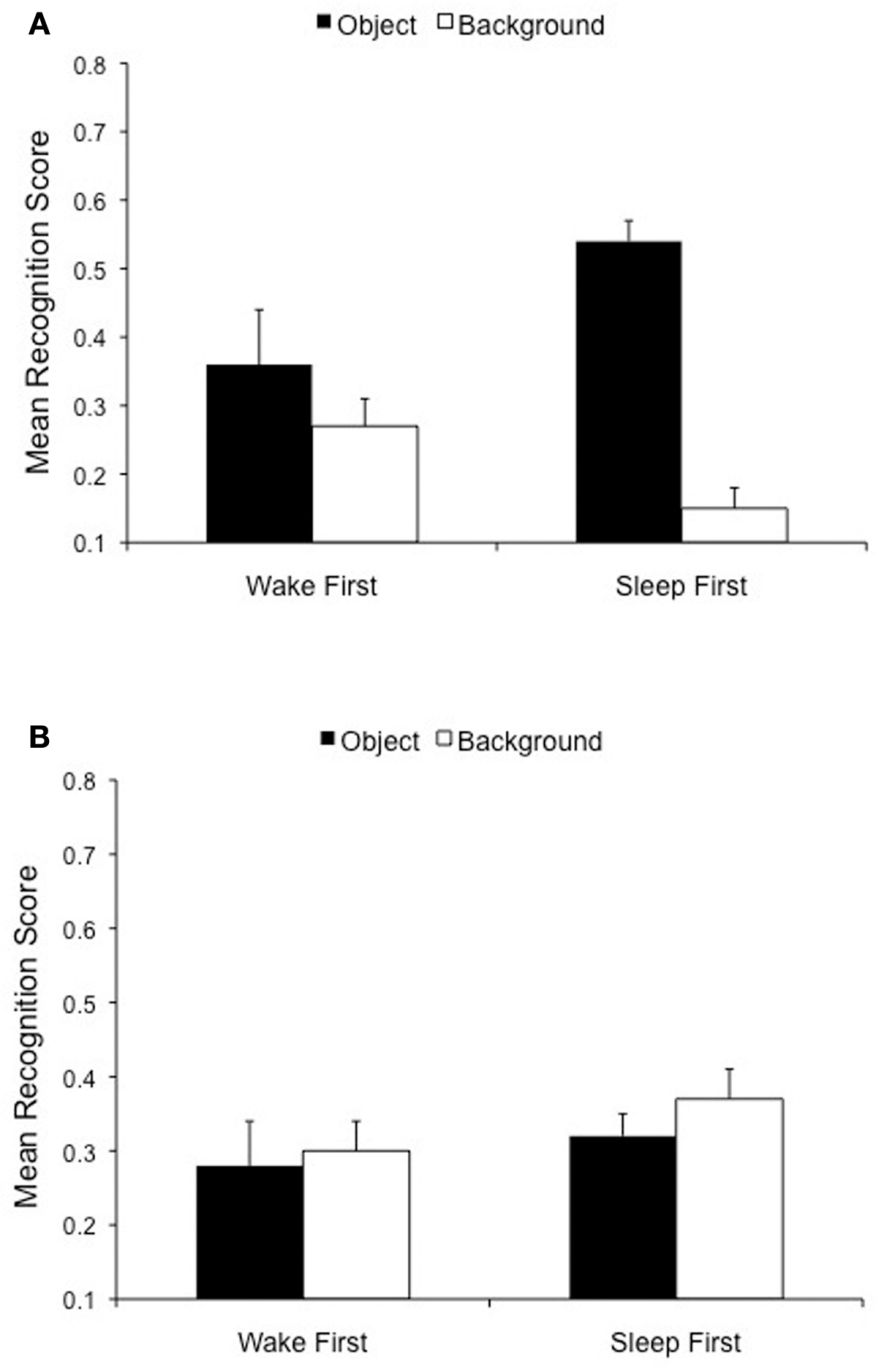
**Twenty-four hours delay memory performance is modulated by the placement of sleep. (A)** Negative objects are better remembered in the Sleep First (*p* < 0.05), compared to the Wake First, condition, but the opposite is true for associated backgrounds (*p* < 0.05). **(B)** The Sleep First and Wake First groups do not differ on memory for any component of the neutral scenes.

Nevertheless, the strength of the trade-off effect is strongly modulated by the nature of the delay between training and test. In the Payne et al. (2008) study, we found evidence for a magnified trade-off following a night of sleep relative to both a 30 min baseline condition and a period of daytime wakefulness. An interference account would deem this effect merely passive and temporary, and would thus predict no differences between the Sleep First and Wake First groups in a memory test given 24 h post-encoding. A consolidation account, on the other hand, would predict a lasting change in the trade-off, and perhaps a magnification of it if sleep triggers changes that continue throughout the following day. To differentiate between these possibilities, we examined whether Object valence (Negative, Neutral) and Scene component (Object, Background) further interacted with the Delay variable (24 h Sleep First vs. 24 h Wake First). There was a highly significant 3-way interaction among the variables, both for Specific recognition, [*F*_(1, 42)_ = 21.3, *p* < 0.001], and Overall recognition [*F*_(1, 42)_ = 18.3, *p* < 0.001], which reveals distinct patterns of emotional remembering in the Sleep First and Wake First conditions (Figure 5). Negative, but not neutral, objects were better remembered in the Sleep First condition than in the Wake First condition. This effect was significant for Specific recognition, [*t*_(42)_ = 2.0, *p* < 0.05], and emerged as a strong trend for Overall recognition, [*t*_(42)_ = 1.8, *p* = 0.08]. Moreover, the backgrounds associated with negative, but not neutral, objects were more poorly remembered in the Sleep First condition than in the Wake First condition, an effect that was significant for both Overall recognition, [*t*_(42)_ = 2.2, *p* = 0.03] and Specific recognition, [*t*_(42)_ = 2.7, *p* = 0.01]. Thus, while negative object memory was enhanced in the Sleep First relative to the Wake First condition, memory for the backgrounds on which they were presented was impaired in the Sleep First relative to the Wake First condition (see Figure 5A).

**Figure 6:**
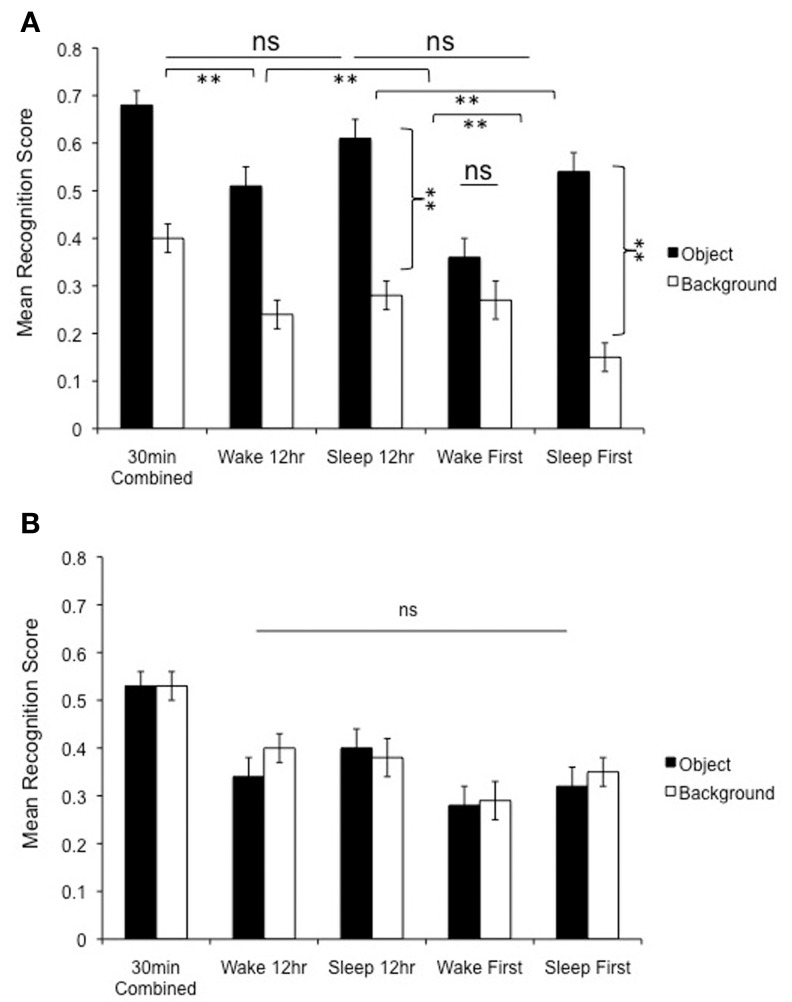
**(A)** Change in memory across time. Relative to the 30 min condition, negative object memory was preserved 12 h later provided that subjects slept in the delay interval (Sleep 12 h group). Moreover, there was no further deterioration in negative object memory over the next 12 h (comparison between the Sleep 12 and 24 h Sleep First group). However, the backgrounds associated with these negative objects continued to deteriorate across these delay intervals, suggesting that sleeping soon after learning is key for protecting memory for emotional components of scenes. In the wake groups, on the other hand, memory for negative objects continued to deteriorate from the 30 min to Wake 12 h and from Wake 12 to 24 h Wake First delays, which depicts a pattern of increasing memory deterioration for negative objects in the wake groups. Note also the absence of the trade-off effect in the 24 h Wake First group (no difference in memory for objects vs. backgrounds), as well as the increased magnitude of the trade-off effect in the two sleep conditions (sleep 12 and 24 h Sleep First—see brackets). ^**^p < 0.01. **(B)** Memory for neutral scenes shows a net decline over time, but note that the groups groups do not differ in memory for objects vs. backgrounds at any of the delay intervals.

In addition, the emotional memory trade-off effect emerged in the Sleep First, but not the Wake First, condition. Although the 2-way interaction between Object Valence and Scene Component was significant in both the Sleep First group, [*F*_(1, 21)_ = 73.3, *p* < 0.0001] and the Wake First group, [*F*_(1, 21)_ = 5.1, *p* = 0.03], only the Sleep First group showed evidence *both* of superior memory for negative over neutral objects, [*t*_(21)_ = 6.3, *p* < 0.0001], and impaired memory for backgrounds associated with negative over neutral objects, [*t*_(21)_ = 5.0, *p* < 0.0001]. The Wake First group, on the other hand, showed evidence only for the former, [*t*_(21)_ = 2.5, *p* = 0.02], but not the latter comparison, [*t*_(21)_ = 0.12, *p* = 0.90, *ns*]. These patterns can be seen in Figure 6. This finding demonstrates that the trade-off effect following a 24 h delay is driven largely by the Sleep First, and not the Wake First, condition.

### Changes in scene memory over time

In a previous study using identical methods (Payne et al., 2008), we examined memory for these scenes across three additional time intervals: 30 min, 12 h spanning a night of sleep, and 12 h spanning a day of wakefulness (Figure 1, labeled “a”). We present this information in relation to the 24-h (i.e., Sleep First and Wake First) delay intervals because it helps us understand how the components of these emotional scenes continue to change over time, and whether sleep has a longer lasting influence on their consolidation. As a caveat, it should be noted that these are cross-experiment comparisons; we are not tracking changes in memory in the same individuals. Still, given the similarity of the samples in the two studies (subjects were university students of the same age), we think these comparisons provide important preliminary evidence for how the components of these emotional scenes are retained over different delays, and can help us understand whether sleep has a longer lasting influence on their consolidation. Although the pattern of findings reported below was similar for all types of memory measured, we report corrected specific memory scores here^1^ because those data (i.e., for the 12 h sleep and wake groups) were not reported in the Payne et al. (2008) study.

A 2 (Object valence: Negative, Neutral) × 2 (Scene component: Object, Background) × 5 (Delay: 30 min, 12 h Sleep, 12 h Wake, Sleep First, Wake First) repeated measures ANOVA conducted on corrected specific recognition memory revealed a highly significant interaction among the three factors, [*F*_(4, 126)_ = 5.4, *p* = 0.001] (Figures 6A,B). Several interesting patterns emerge when comparing the 30 min, 12 h Sleep, and 24 h Sleep First conditions (see Figure 6A). First, there was no impairment in memory for negative objects tested after a 30 min delay vs. a 12 h delay, provided that delay contained sleep (*p* = 0.25); if, however, this delay occurred across a 12 h delay of wakefulness, memory for negative objects suffered significant deterioration (*p* = 0.002). Backgrounds, on the other hand, continued to deteriorate regardless of delay condition (12 h wake, *p* = 0.001, 12 h sleep, *p* = 0.006), suggesting that sleep selectively preserves only the emotional components of these scenes (see Payne et al., 2008). Strikingly, this selective benefit of sleep may last across even longer delays: there was no significant difference in memory for negative objects tested after 12 h vs. 24 h when sleep occurred soon after study (i.e., when comparing the 12 h Sleep and 24 h Sleep First conditions; *p* = 0.20), but there was an additional, precipitous decline in memory for their associated backgrounds that occurred over this additional twelve hour delay (*p* = 0.009).

An interesting consequence of this continued deterioration of negative scene backgrounds is a further magnification of the trade-off in the 12 h Sleep and 24 h Sleep First condition, which can be seen by examining the difference between objects and backgrounds (see curved connectors in Figure 6A). Clearly, sleeping soon after encoding the scenes leads to the largest trade-off effects 12 and 24 h later. In fact, while the trade-off effect is present in the 12 h Wake condition, it dissipates in the 24 h Wake First condition (Figure 6A).

Looking next at the neutral scenes (Figure 6B), one can see that there is little to report, other than a continual decline in recognition rates for both objects and backgrounds across increasing delays. There were no significant differences between objects and backgrounds in any condition, nor were there differences, in either objects or backgrounds, between the 12 h Sleep and 12 h Wake conditions, or the Sleep First and the Wake First conditions. Thus, while the placement of sleep appears very important for selectively remembering the components of emotionally negative scenes, it has no influence on memory for the backgrounds associated with negative objects, or for neutral scenes in their entirety.

## Discussion

The field of emotional memory research is growing rapidly, but there is still much to learn about how memories for emotional events are processed, stored, and how they change over time (Payne and Kensinger, 2010). Although substantial evidence now suggests that the offline brain state of sleep provides ideal conditions for memory consolidation (reviewed by Stickgold, 2005; Ellenbogen et al., 2006; Diekelmann and Born, 2010) and transformation (reviewed in Payne and Kensinger, 2010, 2011), considerably less work has examined sleep's role in emotional memory formation. While several studies have shown that sleep enhances emotional episodic memories over neutral ones, the time course of these effects remains to be fully understood (although see Wagner et al., 2006 for an excellent preliminary study), as do the sleep stages involved.

Here, we show a clear relationship between REM sleep and preferential emotional memory consolidation. Memory for emotional objects (but not neutral objects or the backgrounds associated with either emotional or neutral objects) was positively correlated with REM sleep, but not with any other sleep stage. Indeed, the only correlations to emerge between any of the measures of memory and sleep were those between the two REM measures (time spent in REM sleep and % of the night spent in REM sleep) and correct recognition of emotional objects (for both specific and overall recognition). This finding builds on previous studies using naps (Nishida et al., 2009) and split-night designs (Wagner et al., 2001) to implicate REM sleep in emotional memory consolidation. To our knowledge, however, it is the first to reveal a positive correlation between REM sleep and *selective* consolidation of emotional aspects of scenes in an overnight design. The fact that emotional memory retention correlates with a specific sleep stage reduces concerns that exposure to waking interference in the wake group (rather than active consolidation processes in the sleep group) drives the emotional memory benefit observed in the sleep group. If sleep served to merely passively protect memories from waking interference, one might expect total sleep time to correlate with memory performance, yet that was not the case here. Instead, a particular sleep stage—REM sleep—correlated specifically and exclusively with retention of emotional objects. The specificity of this correlation also reduces concerns that our results are due to time of day influences alone.

Further evidence against an interference interpretation is provided by the 24 h data. By an interference account, sleep passively, and transiently protects memories from retroactive interference, but only until they are exposed to interference the subsequent day (Wixted, 2004). Recently, an “opportunistic theory” of memory consolidation has been posited (Mednick et al., 2011), which argues that *any* condition resulting in reduced exposure to interference will benefit declarative memory consolidation. Thus, sleep *per se* is not uniquely beneficial to memory. However, even in our prior study (Payne et al., 2008), sleep's benefit to memory was strongly modulated by emotionality and scene component; that is, sleep and wake exerted a similar effect on memory for neutral objects and all backgrounds, but gave a specific boost to memory for emotional objects. Thus, while the broad pattern of results reported in that study appear to fit a more opportunistic account of memory consolidation, the dissociable and differential effects of wake and sleep on consolidation of emotional and neutral objects, and backgrounds is not parsimoniously explained by such an account. The current study further rules out an interference interpretation by assessing memory after two 24 h delay intervals, which, critically, contained equal amounts of sleep and wakefulness. In spite of the fact that waking interference was equated in the two groups, participants who slept shortly after learning (24 h Sleep First condition) had superior memory for emotional objects compared to those whose sleep was delayed for 16 h post-encoding following a full day of wakefulness (24 h Wake First condition). Together with the selective REM sleep correlation reported above, it becomes difficult to explain the current data via an interference account alone. Similarly, while we recognize that the 24 h data are subject to circadian confounds, and concede that a time of day explanation cannot be fully ruled out in that design (see Schmidt et al., 2007 for an excellent review of circadian influences on cognition), the selective correlation with REM sleep minimizes concerns about a strict circadian interpretation of our results (although we acknowledge that circadian influences could be operating in concert with REM sleep physiology, especially as chronotype was not controlled for). Given that emotional memory performance has also been associated with REM sleep in a daytime nap study (Nishida et al., 2009), where all subjects were trained and tested in the afternoon, we believe that active (neurobiological) processes occurring during (REM) sleep are likely to play a key role (Ellenbogen et al., 2006; Diekelmann and Born, 2010) in the selective emotional memory consolidation effects observed here.

The 24 h data not only suggest an active sleep-dependent consolidation process, but also emphasize that the *selectivity* of the process is maximized when sleep follows soon after learning. As can be seen in Figure 5, the difference in memory between negative objects and their backgrounds was larger in the Sleep First condition than in the Wake First condition, and only in the Sleep First condition did memory for backgrounds accompanying negative objects fall below the levels of memory for backgrounds accompanying neutral objects. Thus, although sleep conveys a benefit to memory regardless of how soon after learning the placement of sleep occurs, it is the *selectivity* of that benefit that is enhanced when sleep follows soon after learning.

The selective effects of sleep on memory are further emphasized in the comparisons of the 24 h conditions to the 12 h data from Payne et al. (2008). Memory for emotional objects did not deteriorate between the first 12 h (12 h sleep group) and the second 12 h (24 h Sleep-First group), suggesting that sleeping shortly after learning selectively stabilizes the emotional components of scene memories, such that subsequent wakefulness has a diminished negative effect. Notably, this “sleep first” effect has also been seen with tasks examining memory for paired associates (Payne et al., 2012), vocabulary learning (Gais et al., 2006), face-location pairs (Talamini et al., 2008), and an observational learning task (Van Der Werf et al., 2009), and has clear implications for learning (maximizing sleep's benefit by sleeping at the appropriate time after learning, e.g., studying one final time for an exam prior to bedtime). However, the backgrounds associated with the emotional objects suffered profound deterioration, above and beyond that seen after the first 12 h. This suggests that while the sleeping brain apparently “selects” emotional components of scenes for preferential consolidation, a benefit that lasts across a longer (24 h) delay interval, it does not give similar weight to memory for backgrounds, instead letting them continue to deteriorate (or perhaps actively suppressing them).

These findings suggest that the sleeping brain selects for consolidation only what is most emotionally salient about an experience and perhaps most relevant to future goals (Payne and Kensinger, 2010). Previous accounts have conceptualized such preferential emotional remembering as an encoding phenomenon (Loftus et al., 1987; Reisberg and Heuer, 2004; Talmi et al., 2008; Niu et al., 2012). More attention is paid to emotionally salient information at encoding; thus it is better remembered later on. But here we show that post-encoding, sleep-based consolidation processes also play a role in cementing and magnifying the emotional memory trade-off effect. If encoding factors alone were responsible for the trade-off effect, we would expect memory for scene components to be identical in the two delay conditions. If, on the other hand, sleep-based consolidation processes influence the development of the trade-off effect, then we might expect a divergence in the scene components in the two conditions, perhaps with a greater magnification of the trade-off in the Sleep First group than in the Wake First group, which is exactly what we find here. Between the 12 and 24 h delay groups, the trade-off was magnified across time when sleep occurred soon after learning. However, if wake occurred first in this 24 h interval, the trade-off that was observed even in the 12 h wake group becomes non-existent. This finding provides strong evidence that sleep-dependent consolidation processes are critical for the development and maintenance of the trade-off effect, not exclusively processes that occur during encoding.

A number of other studies support this notion of sleep continuing to protect and promote emotional memory processing over the long-term (Wagner et al., 2006; Sterpenich et al., 2009). Wagner et al. (2006) found that memory for emotional, but not neutral, narrative descriptions persisted 4 years later in subjects who had slept shortly after learning as compared to those who first remained awake. A potential mechanism for such effects is the refinement and redistribution of these memories to different regions of the brain, which sleep appears to support more effectively than wakefulness (Payne and Kensinger, 2011), and which results in changing activation patterns as delays grow longer (Sterpenich et al., 2007, 2009). For instance, Sterpenich et al. (2007, 2009) found that remembering emotional pictures studied before a period of sleep transferred activation from the medial temporal lobe to the cortex during testing occurring 3 days to 6 months after learning, a pattern of activation that was much weaker in those who had remained awake after learning. Thus, there is compelling evidence that sleep, but not wake, soon after learning acts at the neural level to efficiently consolidate emotional memories, and these effects remain visible months and even years later.

An alternative interpretation of these and other sleep data is provided by the synaptic homeostasis hypothesis (Tononi and Cirelli, 2003). According to this hypothesis, learning increases the strength of synapses in the brain, which in turn requires synaptic downscaling during sleep, and specifically slow wave sleep, to return synaptic strength to baseline levels (Tononi and Cirelli, 2003). By this account, stronger memories are preserved because even after downscaling, these memories persevere the reduction in synaptic strength. Our results could be broadly consistent with this theory insofar as we find a degradation of memory over time for all scene elements, but with the least degradation for the most salient aspects of the scenes, the emotional objects. However, rather than observing a positive correlation between memory enhancement and SWS, as would be predicted by the synaptic homeostasis hypothesis, we find that emotional memory enhancement is correlated with REM sleep. While our results are not easily explained by synaptic homeostasis, and instead appear to be the result of active consolidation processes occurring during specific sleep stages, the two accounts are not mutually exclusive. It may well be that some refinement of memory traces occurs during slow wave sleep, which is in turn followed by REM sleep-based processes that are key for emotional experiences.

Collectively, these results have important implications for adaptive remembering. It has long been known that forgetting is adaptive, but optimal memory functioning demands that we maintain a record of the events that hold future relevance (Levine and Edelstein, 2009). It thus seems reasonable that sleep-specific processes would selectively preserve the most salient, negative aspects of the scenes used here, even if this comes at the cost of the peripheral information, because knowledge of the emotional component will provide a greater benefit to the individual in the future. However, preferential retention of emotional information may also come at a cost; rumination and flashbacks may reflect vivid memories of negative events that are removed from the context in which they occurred, and as such, the present research may have implications for the etiology and prognosis of diseases like depression and post-traumatic stress disorder (PTSD). Both disorders are associated with sleep disturbances and abnormal sleep architecture, with several studies pointing to specific disruptions in REM sleep (Berger and Riemann, 1993). In fact, recent studies suggest that those with a history of trauma but not current PTSD do not exhibit the typical emotional memory trade-off, instead showing an overall memory bias for both the emotional and neutral objects at the expense of the backgrounds associated with both stimulus types (Mickley Steinmetz et al., 2012). Investigating what makes these individuals different from traumatized individuals who do develop PTSD, and what role sleep may play in this relationship, will be important topics for future study.

Understanding the cognitive and neural mechanisms underlying the development, maintenance, and long-term consequences of emotional memory trade-offs, and how sleep is involved, is critical for knowledge of healthy emotional processing as well as its dysfunction. The present data contribute to that larger goal by showing that sleep soon after learning triggers lasting memory for negative emotional components of an experience, at the expense of neutral components. The findings reported here cannot be easily accounted for by an interference account, and instead reveal that selective emotional remembering is associated with active processing during REM sleep.

### Conflict of interest statement

The authors declare that the research was conducted in the absence of any commercial or financial relationships that could be construed as a potential conflict of interest.

## Footnotes

^1^False alarms, i.e., “new” responses to “same/identical” items, were subtracted from the specific memory hit rate. We refer the interested reader to the Payne et al. (2008) study for information on overall recognition memory following the sleep and wake delays. Because those data were previously published, we do not report them here. We note, however, that the ANOVA referred to above (i.e., with the 5-level Delay variable), when conducted on corrected overall recognition, returned a similarly significant interaction among the 3 factors, [*F*_(4, 126)_ = 5.7, *p* < 0.0001].
